# Learning across diverse biomedical data modalities and cohorts: Challenges and opportunities for innovation

**DOI:** 10.1016/j.patter.2023.100913

**Published:** 2024-01-17

**Authors:** Suraj Rajendran, Weishen Pan, Mert R. Sabuncu, Yong Chen, Jiayu Zhou, Fei Wang

**Affiliations:** 1 Tri-Institutional Computational Biology & Medicine Program, Cornell University, Ithaca, NY, USA; 2 Division of Health Informatics, Department of Population Health Sciences, Weill Cornell Medicine, New York, NY, USA; 3 School of Electrical and Computer Engineering, Cornell University, Ithaca, NY, USA; 4 Cornell Tech, Cornell University, New York, NY, USA; 5 Department of Radiology, Weill Cornell Medical School, New York, NY, USA; 6 Department of Biostatistics, Epidemiology, and Informatics, University of Pennsylvania, Philadelphia, PA, USA; 7 Department of Computer Science and Engineering, Michigan State University, East Lansing, MI, USA

## Abstract

In healthcare, machine learning (ML) shows significant potential to augment patient care, improve population health, and streamline healthcare workflows. Realizing its full potential is, however, often hampered by concerns about data privacy, diversity in data sources, and suboptimal utilization of different data modalities. This review studies the utility of cross-cohort cross-category (C^4^) integration in such contexts: the process of combining information from diverse datasets distributed across distinct, secure sites. We argue that C^4^ approaches could pave the way for ML models that are both holistic and widely applicable. This paper provides a comprehensive overview of C^4^ in health care, including its present stage, potential opportunities, and associated challenges.

## The bigger picture

Machine learning and artificial intelligence offer immense potential to improve health care in the future, but a variety of real-world issues present hurdles to adoption. Data must be handled carefully to protect patient privacy. Data collected from different sources can vary in quality and completeness. Data from different assay methods (i.e., multimodal data) can be hard to combine and integrate. Here, we review and discuss methods, models, and workflows that are well suited to handling these kinds of challenges, which we argue will ensure that the promise of machine learning for health care is fully realized.

## Teaser

Machine learning has great potential to revolutionize health care. Yet, it is hindered by privacy concerns, varied data sources, and limited use of different types of data. Cross-cohort cross-category learning (C^4^) aims to overcome these obstacles by merging varied datasets. C^4^ involves multiple secure locations, each contributing different data types, to build comprehensive machine learning models. These models utilize data from every site, offering a broader understanding. This review explores the applications and challenges of C^4^ in health care.

## Introduction

Machine learning (ML) in health care is a rapidly evolving field, presenting numerous opportunities for progress. Active and passive patient data collection, both during and outside medical care, can be utilized to address health challenges. As a result, ML has become an essential tool for processing and analyzing these data in various domains, including natural language processing, computer vision, and more. ML systems have demonstrated their potential to enhance patient experiences, improve population health, reduce per capita healthcare costs, and optimize healthcare providers’ workflows.^1^^,^^2^^,^^3^^,^^4^ However, the real-world clinical and cost benefits of ML in health care remain limited, indicating a significant gap in its application.

Data privacy is a major challenge for the application of ML in health care because it restricts the potential for pooling together sensitive data such as the electronic health record (EHR) from multiple sites. Federated learning (FL) offers a promising approach to addressing this issue by enabling the aggregation of fragmented, sensitive data from various sites without sharing the raw data.^5^^,^^6^^,^^7^ In brief, a typical FL architecture consists of a central aggregator designed to obtain global ML model parameters by iteratively exchanging their local updates, and FL typically assumes that the data at different sites share the same information modalities.^8^^,^^9^^,^^10^

Almost all healthcare problems involve multiple different data modalities, such as EHR, medical imaging, and genetic sequencing. Methods and systems that integrate multiple modalities are becoming more and more important.^11^^,^^12^^,^^13^^,^^14^^,^^15^ Multimodal learning (MML) refers to such a paradigm aiming at integrating disparate data modalities to capitalize on complementary information, thereby improving performance. Existing MML approaches typically assume that the different data modalities are available for the same sample cohort—e.g., for each patient, we have his or her EHR and medical image,^16^^,^^17^ and the research focus is how to align the different data modalities and combine them together.

**Figure 1:**
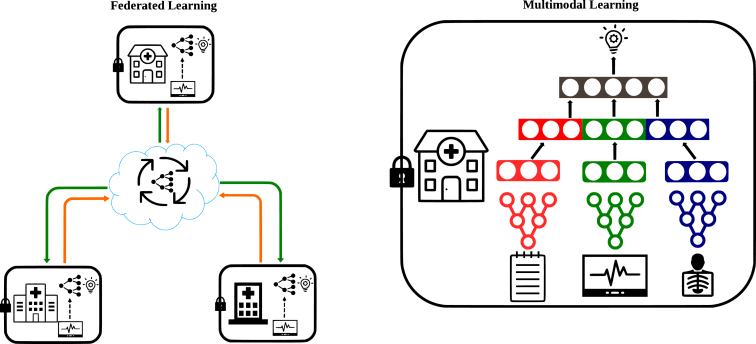
Federated and Multimodal Learning Federated (left) and multimodal (right) learning diagrams.

Despite the existing research and reviews on FL and MML (whose schemas are summarized in Figure 1), in reality we are usually faced with the scenario that different biomedical datasets include sample cohorts with different information modalities. Learning in such a scenario, which is referred to as cross-cohort cross-category learning (C^4^), can integrate more comprehensive information compared to FL and MML, and thus make the model more comprehensive and robust. In this scoping review, we cover several current implementations of C^4^, discuss the opportunities that C^4^ can bring to health care, point out potential challenges, and summarize future research directions.

### C^4^ learning

**Figure 2:**
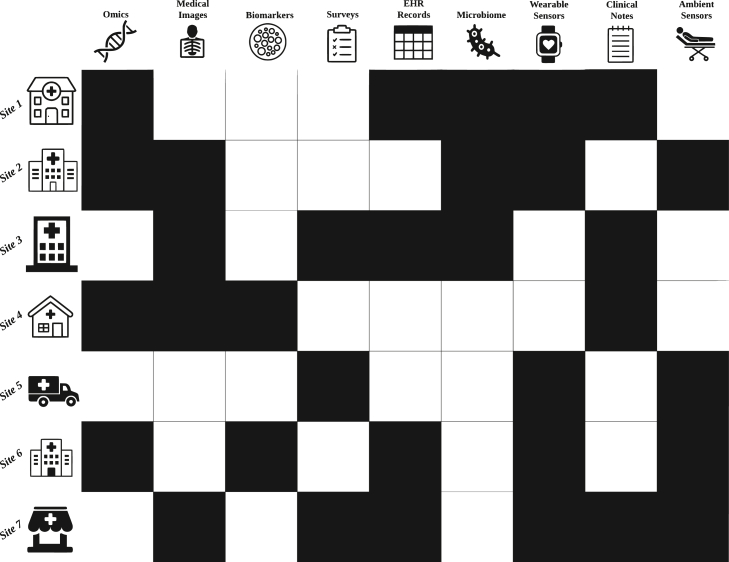
Overview of an C^4^ setting in health care Black squares are client modalities that are available for use in model generation.

C^4^ aims at integrating information from disparate datasets (residing at different sites) composed of different data modalities. Different datasets may have different information modalities, resulting in a patched setting (Figure 2). Although there are many existing statistics and ML approaches for missing value imputation,^18^^,^^19^^,^^20^ they have mostly focused on the single modality setting and the missingness could be at or not at random across different feature variables. In the C^4^ setting, certain datasets can miss certain information modalities entirely, and there could be distribution shift across different datasets due to sample heterogeneity. In addition, there could be privacy concerns, so different sites may not be able to share their own data with other sites, which makes the learning process more challenging.

**Figure 3:**
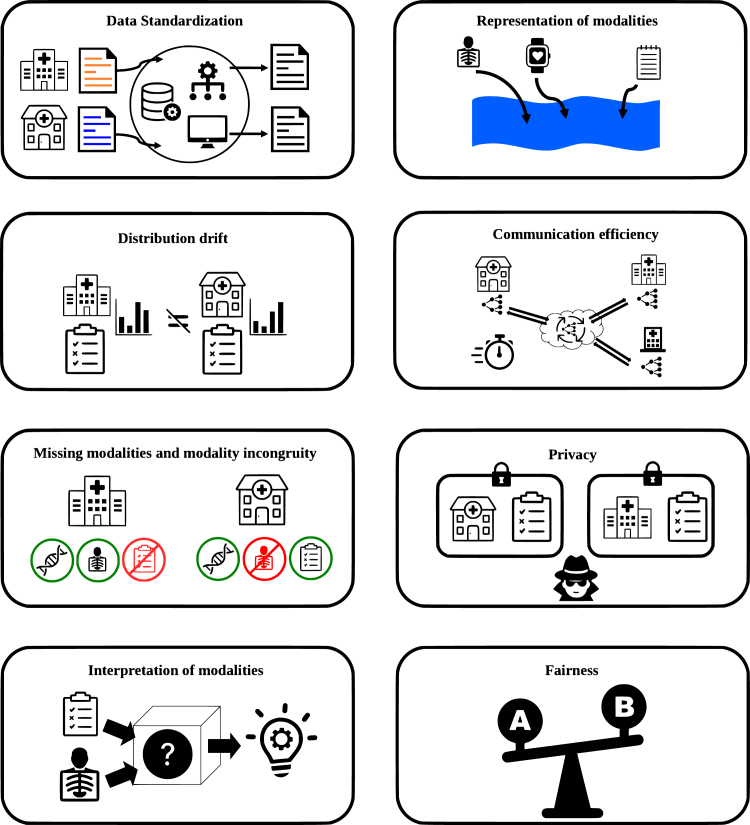
Challenges associated with C^4^ Diagram showing the several challenges surrounding developing models within the C^4^ setting.

Building learning algorithms within the C^4^ setting has the potential to advance health care in several ways. As previously mentioned, models developed at one site are not readily applicable to another site due to data heterogeneity. Incorporating various data modalities across different sites can mitigate some biases that currently affect ML models. Furthermore, in real-world scenarios, certain organizations may not have access to multiple modalities. Secure information sharing across sites can facilitate the development of robust models capable of integrating multiple modalities of healthcare data, even at organizations lacking those modalities. A method built with the C^4^ setting in mind can prove helpful in situations where different sites have related but distinct tasks, as it may be possible to train a shared model backbone for downstream tasks. The backbone model can be pretrained using all of the available modalities and patients across different sites. The pretraining process can be implemented using a general-purpose approach, such as employing a self-supervised strategy, which facilitates the learning of informative representations applicable to a wide range of downstream tasks. After training the backbone model, it can be fine-tuned to cater to specific tasks at each site.

### Current implementations of C^4^

In this subsection, we summarize a few existing C^4^ implementations in the literature.

Federated transfer learning (FTL) is a unique variation of FL that utilizes distinct datasets, which differ in both sample and feature space. FTL offers users an effective way to manage disparities in data distribution across clients.^21^^,^^22^ A core component of FTL is transfer learning, a ML technique that aims to enhance the performance of target models developed on target domains by reusing the knowledge contained in diverse but related models developed on source domains. FTL can be performed in multiple ways, but generally, knowledge across sites is securely transferred and/or aggregated, despite differing feature spaces between sites. Overall, there are two strategies in FTL: (1) using pretrained models in related tasks or (2) using domain adaptation to transfer knowledge from a source domain to a related target domain. Chen et al. developed an FTL algorithm, FedHealth, which uses domain adaptation to analyze multimodal healthcare data from wearables. To address the data isolation and heterogeneity issues associated with wearable data, FedHealth first trains a model on public data at the central server, which it then transfers to clients iteratively for further personalization.^23^ To apply FTL within C^4^ settings, the relationships between modalities can be regarded as the knowledge to be transferred across sites. Following FedHealth’s methodology, models capable of inferring missing modalities can be trained on public data and adapted to each site. A crucial aspect of this approach is ensuring that the public data encompass a comprehensive range of potential modalities across all sites.

As previously discussed, clients in a C^4^ setting are likely to lack all data modalities at their site, which constrains their ability to develop integrated models. Confederated learning provides a solution to this issue.^24^ In confederated learning, ML models are trained on data distributed across diverse populations and data types, employing a three-step approach. The concept was introduced in a study where a patient population’s data were split both horizontally and vertically—i.e., different sites had varying combinations of data (diagnostic data, medications, lab tests) and patients. Notably, confederated learning requires an auxiliary dataset to be available at the central server, which may not be realistic in real-world scenarios. To perform confederated learning, conditional generative adversarial networks with matching loss (cGAN) were trained using data from the central server to infer one data type from another. These cGANs are transferred to each local site, where the missing data types are imputed with generated samples. Thereafter, task-specific models, such as diagnosis prediction, were trained in a federated manner (e.g., federated averaging) across all of the sites simultaneously. Compared to other methods, confederated learning is simple to implement and does not require any patient ID matching. A core weakness, however, is that the success of the approach depends on the quantity and heterogeneity of data available at the central server. The performance of the subsequent confederated model can be affected by any discrepancies between the auxiliary data and the data at each local site.^24^

In a C^4^ setting, even when all of the modalities are available, it is essential to effectively integrate these modalities for modeling downstream tasks. Several studies have explicitly combined FL and MML to develop federated multimodal learning (FML) systems.^25^^,^^26^^,^^27^ These systems are designed to integrate multiple data modalities across sites. Some personalized recommendation systems use FML with multimodal matrix factorization methods to provide privacy-preserving predictions based on both text and demographic data.^28^^,^^29^ Salehi et al. propose FLASH, which fuses data from light detection and ranging (LIDAR), global positioning system (GPS), and camera images to train a federated model across vehicles, optimizing vehicular communication transmissions.^30^ Another FML method, FedMMTS, uses multimodal analytics to create privacy-preserving systems that enable autonomous decision making for vehicles in a simulated environment.^31^ Although FML systems have been applied in multiple domains, their implementation in health care has been limited. Challenges associated with missing data, patient privacy, and the need for clinical interpretability constrain the adoption of FML in health care. Che et al. designed H-FedMV and S-FedMV, which perform FML across sites using federated averaging, the latter of which is able to account for sequential information within medical data. Modalities that were integrated include textual and time-series data, both of which were used to diagnose patients with bipolar disorder.^32^ Another study utilized an FML system to predict oxygen requirements for coronavirus disease 2019 (COVID-19) symptomatic patients by combining data from EHRs and chest X-rays. To integrate the different modalities and increase the interaction between data types, a Deep & Cross network architecture was used across all of the sites, followed by fully connected layers for performing prediction. To add privacy-preserving measures to their model, differential privacy was implemented in the federated weight-sharing mechanisms. Although the model performed relatively well on validation data, the architecture required the presence of all of the modalities at all of the sites, which is unrealistic in a real-world scenario without losing a considerable amount of available data.^33^

The aforementioned methods can be suitable for the C^4^ setting, where the goal is to learn from multiple data modalities distributed across secure silos. Each method possesses distinct weaknesses and strengths, which we will examine in the following sections. However, these approaches collectively demonstrate the potential for advancing the development of robust and generalizable ML models in the healthcare domain.

## Opportunities for solutions in C^4^ settings

### Predictive diagnosis and risk prediction

Predictive analytics has emerged as a valuable tool in medical decision making because patients’ responses to treatments, particularly for chronic diseases, can vary significantly. ML algorithms, using data and outcomes from past patients, can offer insights into the most effective treatment methods for current patients. Most cutting-edge clinical risk predictive models are based on deep learning and trained end-to-end. However, the robustness of diagnostic or risk prediction tools relies heavily on the breadth of data used to build them. Diagnostic ML models developed using site-specific unimodal data often face challenges when adapting to other clinics.^34^^,^^35^^,^^36^ This issue persists even when incorporating federated methods due to heterogeneity and lack of local personalization.^37^^,^^38^

The integration of multiple modalities has improved the performance of these algorithms across different sites. For example, stroke manifestations can be found in both EHR and medical imaging data, indicating that combining both could result in more accurate risk prediction models. Boehm et al. used a multimodal dataset, including computed tomography (CT) scans, H&E-stained pathology slides, omics, and clinical data, to stratify risk for patients with high-grade ovarian cancer. This approach revealed the complementary prognostic information provided by each modality.^39^ Ali et al. combined data from sensors and EHRs to detect cardiovascular disease while generating automated recommendations for patient care.^40^ However, medical data are often siloed, making it difficult to access large multimodal datasets. Qayyum et al. introduced clustered federated learning (CFL), an algorithm that uses a federated multitasking framework to group clients into modality-dependent clusters with jointly trainable data distributions for COVID-19 diagnosis prediction. CFL outperformed other unimodal federated models.^41^ Another study developed a generalized federated multimodal disease diagnosis prediction model using a fusion and least-squares algorithm, which significantly outperformed locally trained unimodal counterparts.^42^ Agbley et al. employed a multi-modal federated learning (MMFL) framework to create a melanoma detection model using matched EHR data and skin lesion images.^43^ Alam et al. developed FedSepsis, a model for early detection of sepsis that incorporated tabular and textual data from EHRs using FML. The study used low-computational edge devices, such as Raspberry Pi and Jetson Nano, to address practical challenges.^44^

Models developed for C^4^ have demonstrated comparative advantages over both unimodal federated models and multimodal single-institution models for risk prediction. Although heterogeneity across silos still needs to be addressed when developing models in a C^4^ setting, these studies have shown clinical potential in terms of performance and interpretability.

### Personalized omics for precision health

The advent of next-generation sequencing technologies has led to increased interest in studying human health by interpreting molecular intricacies and variations at multiple levels, such as the genome, epigenome, proteome, and metabolome. These omics data integrate large amounts of personalized patient data, which is crucial for understanding individual disease states, distinguishing subphenotypes, and developing digital twins, among other applications.^45^^,^^46^^,^^47^ ML methods offer innovative techniques for integrating various omics data to discover new biomarkers, potentially aiding in accurate disease prediction and precision medicine delivery. The study of integrative ML methods for multiomics data enables a deeper understanding of biological systems during normal physiological functioning and in the presence of disease, supporting insights and recommendations for interdisciplinary professionals. Shen et al. recently introduced a method to capture and examine multiple molecular data types from a mere 10-μL blood sample, including thousands of metabolites, lipids, cytokines, and proteins, further enriched by physiological data from wearables.^48^ Nonetheless, the study’s limitations stem from its small dataset, potentially affecting its representativeness and statistical robustness. C^4^ could address these limitations by collecting data from various sites and integrating it, allowing for a larger, more representative sample size.

Automated pipelines such as GenoML, developed by Makarious et al., enable users to analyze multiomics data in combination with clinical data while providing a federated module for basic privacy-preserving omics analysis across data silos.^49^ Many current multimodal methods incorporating omics data use matrix factorization methods or canonical correlation analysis (CCA) to combine information from multiple modalities.^42^ LungDWM uses multiomics data to diagnose lung cancer subtypes by fusing omics-specific features extracted from an attention-based encoder. Missing omics-specific features are imputed through generative adversarial learning.^50^

Currently, no platform can efficiently integrate clinical, multiomics, and other data modalities while simultaneously enabling effective management of data analytics accessible to physicians.^51^^,^^52^ Successful integration of different omics data with other data types, such as EHRs and medical images, has the potential to enhance our understanding of a patient’s health, allowing for the development of personalized preventive and therapeutic interventions. Such integrations require big data platforms or methodologies that facilitate the fusion of heterogeneous modalities from multiple silos while allowing real-time care.^53^

### Digital clinical trials

The ever-increasing sources of clinical data from EHRs, claims, and billing data have generated massive amounts of real-world data (RWD) with the potential for translational impacts on patients. In recent years, trial emulation, the process of mimicking targeted randomized controlled trials (RCTs) with RWD such as electronic health records, has gained attention in the medical community. Although RWD is more representative of real patient populations, numerous challenges are associated with conducting trial emulation, such as identifying and controlling confounding variables, constructing proper RCT designs, and determining appropriate causal inference methods for outcome estimation. Although there is a growing body of research addressing these challenges, data access remains a significant limitation, especially for trial emulations focused on less common conditions and treatments. Gaining access to RWD can be a lengthy and costly process, and due to privacy concerns, aggregating private health data, which is often richer in information on specific conditions, can be difficult. Accessing RWD from various clinical sites can help combat data heterogeneity in patient populations, allowing trial emulation hypotheses to be generalized across demographic and geographic groups.^54^ Liu et al. introduced the distributed algorithm for fitting penalized (ADAP) regression models to integrate patient-level data from multiple sites, studying risk factors for opioid use disorder. To securely share information and mitigate heterogeneity across multiple sites, collaborating sites only share first- and second-order gradients when conducting trial emulation.^55^

Integrating data from wearable technologies can also improve trial emulation outcomes. Readings from wearables, such as sleep, physical activity, vital signs, and questionnaires, can provide valuable information for balancing confounders during trial emulation pipelines, despite being noisy. ML techniques can be employed to integrate data from wearables, omics, EHRs, and medical images for digital clinical trials.^56^^,^^57^^,^^58^ Currently, the performance of multimodal trial emulation pipelines is restrained by the lack of compatibility among RWD databases. Moreover, architectures that can concurrently utilize longitudinal RWD from multiple modalities are still not in existence.^59^ Despite the high cost of conducting clinical trials, causal inference using C^4^ can help identify pertinent medications or treatments through trial emulation. By integrating heterogeneous sources of data, both in terms of features and samples, confounding variables can be controlled, enhancing the capabilities of digital clinical trials. SurvMaximin is one such algorithm in this field that combines multiple prediction models from different source outcomes in a federated manner for predicting survival outcomes.^60^

As the costs of real-world clinical trials continue to rise, computational tools will be essential for supplementing hypothesis generation. Confounding patient and environmental variables, spread across multiple data modalities, must be accounted for even in extensive collections of RWD. C^4^ formulations can offer unique ways to mitigate confounding variables and integrate private data sources, enabling hypothesis generation for rare medical conditions.

### Remote monitoring

Medical Internet of Things (MIoT) devices, such as wearables and mobiles, facilitate real-time monitoring of vital physiological parameters and behaviors.^61^ Data such as heart rate, blood pressure, body temperature, blood oxygen saturation, posture, and physical activities are acquired through tools like electrocardiograms (ECGs) and ballistocardiograms (BCGs). These data aid in preventing health issues and predicting COVID-19 symptoms remotely.^62^ Monitoring mental conditions via wearables, which detect key physiological markers, is an evolving field. For example, Xu et al. devised FedMood, a system using mobile phone keystrokes and accelerometer data for depression detection.^63^ Fed-ReMECS fuses various signals for real-time emotional state classification.^64^ Liang et al. developed a privacy-focused multimodal model for mood assessment, surpassing unimodal models in performance.^65^ By facilitating the early identification of health irregularities, wearable technology can also enhance the efficiency of patient management in hospitals. Wireless communication in wearable techniques enables researchers to design a new breed of point-of-care (POC) diagnostic devices.^66^^,^^67^^,^^68^

However, processing information from wearables can be challenging. Data from wearables are intrinsically multimodal, ranging from audio and images to time-series data. Although efforts have been made to fuse data types from various sensors, there are further advantages to combining wearable data with formalized clinical data, such as those recorded in EHRs. Wang et al. proposed an architecture design for COVID-19 diagnosis using a combination of demographic information, medical record text data, patient mobile data, and image data stored across different nodes. While not implementing this design, they highlight the advantages of enabling such architectures for real-time pandemic monitoring.^69^

Another issue with wearable information is data privacy. Healthcare data from different people with diverse monitoring patterns are difficult to aggregate together to generate robust results. Patient confidentiality and data security are major concerns when using wearable devices because ensuring compliance with Health Insurance Portability and Accountability Act (HIPAA) regulations can be challenging. The use of FL could mitigate several of these privacy challenges. Chen et al. extended FedHealth to develop FedHealth 2, which creates personalized models for each client by obtaining client similarities using a pretrained model and then averaging weighted client models while preserving local batch normalization. FedHealth 2 showed increased performance in activity recognition compared to other federated methods.^70^ The Federated Multi-task Attention (FedMAT) framework, built on multimodal wearable data, outperforms baseline methods in human activity recognition and is rapidly adaptable to new individuals. The framework uses an attention module at each client to learn both client-specific features and globally correlated features while ensuring data security.^71^ Reddy et al. propose a blockchain-based FL system using multimodal wearable data to predict COVID-19, enabling relatively secure transmission of pertinent model development information.^62^

Given the multimodal and secure nature of wearable sensor data, there is a direct need for the development of algorithms for C^4^. Through the use of such algorithms, wearable data can be employed for proper remote monitoring, thereby improving patient care.

## Challenges

Implementing C^4^ systems comes with significant challenges (Figure 3). In this section, we discuss some of the key challenges associated with these systems and explore potential solutions and future directions for addressing these challenges.

**Figure 4:**
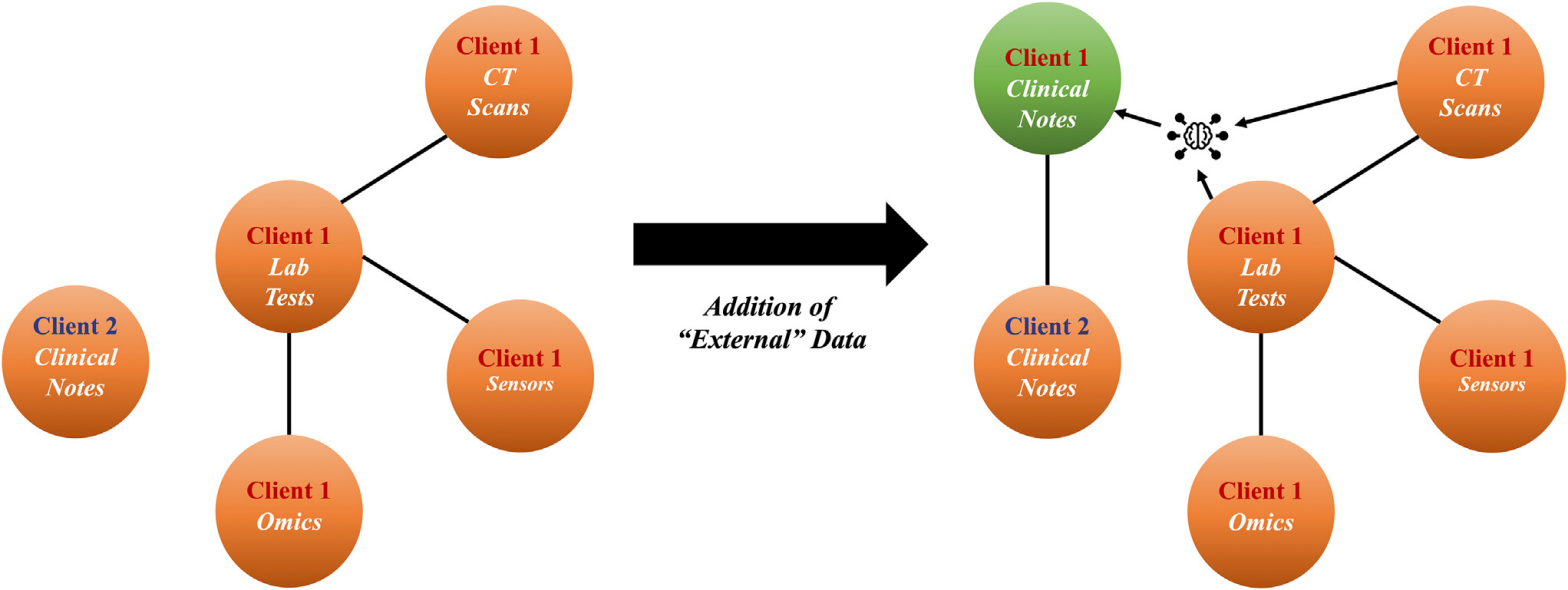
Utilizing external data for connective blocks scenario Lines represent modalities that are the same or are intraclient. Client 1 and client 2 aim for collaborative model generation. Lacking clinical notes, client 1 leverages LLMs, foundation models, biomedical knowledge graphs, and existing modalities to generate a “clinical notes” modality (green), aligning with client 2’s data modality for collaborative model creation.

### Data standardization: Normalizing the data elements across different cohorts

One crucial step before formulating a solution for C^4^ is the data standardization across different cohorts. For example, the terminology coding system (e.g., for diagnosis, medications, procedures, etc.) used in one EHR cohort could be different than the one used in another EHR cohort. In this case, it is important to map them to a common set of data elements, which is typically referred to as common data models (CDMs). In clinical informatics, several CDMs have been developed for normalizing the data elements in EHRs from different consortiums, such as the Observational Medical Outcomes Partnership (OMOP) developed from the Observational Health Data Sciences and Informatics (OHDSI), and the PCORNet CDM developed from the Patient Centered Outcomes Research Institute (PCORI).^72^ Because there are multiple distinct modalities involved within the C^4^ paradigm, it is critical to expand these CDMs across those different modalities. This involves not only the normalization of the terminology of the data elements but also the relationships among them because most of the CDMs are ontologies themselves. For instance, OMOP could be enhanced by introducing modular data structures that cater specifically to diverse data modalities inherent in a C^4^ setting. Furthermore, the metadata descriptors within OMOP might need to be enriched to provide a clearer context for multimodal datasets, making cross-cohort connections more transparent. Recent developments such as ATRACTion OMOP-CDM have taken steps to integrating omics data into the OMOP model.^73^

### Representation of modalities: Effective representation of modalities is necessary for data integration and downstream tasks

With the diversity of healthcare data modalities, integrating the information concisely to allow for optimal model development is essential. Multimodal representation involves the efficient and meaningful vector or tensor presentation of multivariate data, which often features both redundant and supplementary information. Representation can be broadly categorized as either joint or coordinated. The former projects data from various modalities into a shared space, frequently utilizing fusion techniques. Meanwhile, the latter allocates each modality into unique yet coordinated spaces via similarity measures.^74^ Currently, many MML architectures utilize fusion as a way of joining information from multiple modalities.^33^^,^^75^ In the C^4^ context, three principal fusion strategies for handling multimodal data arise. Early fusion merges features from different modalities before main model processing, making it effective when there is strong correlation between data modalities across silos. However, it assumes consistent modality availability, which might be a challenge in C^4^’s diverse data landscape. Late fusion processes each modality separately and combines them toward the end. This offers adaptability within C^4^, especially with missing data, but might overlook subtle intermodality relationships. Hybrid fusion, combining both strategies, is a versatile solution for C^4^. It ensures depth in data interpretation while remaining adaptable, but its complexity may grow with more data and clients. Recent innovations such as the OpenAI Contrastive Language–Image Pretraining (CLIP) highlight the necessity of merging modalities for optimal ML performance. CLIP is a powerful approach that uses a joint representation learning framework to learn a wide array of visual models. The CLIP model is pretrained on a large-scale dataset of images and their associated natural language descriptions, which allows it to learn a rich and robust multimodal representation that captures both visual and textual information.^76^ Multimodal large language models (LLMs) also present novel ways to represent various modalities jointly. PALM-E, a multimodal model developed by Google Research, encodes RWD into a language-embedding space, allowing it to integrate various types of information from sensors. PALM-E has been used to enable effective robot training and build state-of-the-art generalized visual-language models.^77^ However, while showing promise in nonspecific domains, MML fusion models still face challenges in health care, such as being prone to overfitting.^78^ To address this issue, HuggingGPT was developed, which takes advantage of the hundreds of specialized models publicly available on the Hugging Face Hub. HuggingGPT uses an LLM as a central manager that distributes subtasks to several downstream multimodal models to complete an overall objective. Although rudimentary, these objectives can easily scale in complexity as long as certain specialized models exist downstream that are able to perform the subtasks.^79^ In a similar vein, foundation models have gained traction due to their capability to learn a wealth of information from large-scale datasets. These models, pretrained on massive corpora, effectively serve as feature extractors for multiple modalities. Given their expansive knowledge base, foundation models can provide high-level feature embeddings which, when fine-tuned, are attuned to specific downstream tasks.^80^ Furthermore, when dealing with diverse modalities, the embeddings from these models can be used as a common foundation. Techniques such as CCA or mutual information estimation can further align the extracted features from different modalities into a unified representation space. This alignment provides a coherent foundation for subsequent tasks. Since 2022, several medical foundation models have been developed, including holistic artificial intelligence in medicine (HAIM), RETFound, and CheXzero.^81^^,^^82^^,^^83^ However, constructing medical foundation models for the C^4^ context is challenging due to the limited availability of extensive healthcare multimodal datasets.^84^ Additionally, training these models in a C^4^ framework presents privacy concerns because they must generalize across diverse modalities while learning across siloed cohorts.

In a C^4^ setting, colearning methods might be preferable due to their ability to function relatively well in scenarios where modalities may be missing during training or inference.^85^^,^^86^^,^^87^ Colearning uses knowledge transfer from one modality to learn about a less-informed modality. Colearning methods include the utilization of multimodal embeddings, transfer learning, multitask learning, and generative networks, with each method aiding in mitigating real-world issues with multimodal data, such as missing modalities, noisy labels, and domain adaptation.^88^

### Missing modalities and modality incongruity: Solutions need to account for both random and nonrandom missing data modalities

The patchwork characteristic frequently associated with C^4^ scenarios is due to the disparate availability of data modalities across various healthcare sites. Many multimodal learning models typically assume that modalities are complete, meaning they are fully paired during both training and inference. Yet, due to constraints such as privacy or budget, this assumption may not align consistently with real-world conditions. In fact, missingness is often nonrandom in healthcare datasets, with certain sites lacking specific data modalities due to infrastructure. Furthermore, different clinical workflows for acquiring data can also lead to nonrandom missingness across sites. Many investigations have explored novel methods to tackle missingness during inference time.^75^^,^^86^ Although less so, there have also been algorithms proposed for combating missingness in modalities during training. Recently, generative networks such as variational autoencoders, generative adversarial networks, and Long Short-Term Memory networks (LSTMs) have been deployed to generate absent modalities. By learning the joint distribution of multimodal data, these networks can create one modality from another, thereby capturing semantic correlations between modalities.^89^^,^^90^ Although some of these generative methods operate in an unsupervised manner, research indicates the benefits of integrating ground-truth labels through classification loss, specific types of adversarial loss, or triplet loss to more effectively generate modalities.^90^^,^^91^^,^^92^ Lee et al. compared the performances of an LSTM and autoencoder architecture for generating audio modality from red, green, and blue (RGB) images. Their investigation found that the incorporation of classifier loss enhanced the results of the autoencoder-based approach.^93^ Confederated learning uses centrally trained generative networks to combat missing modalities at local sites.^24^ With the recent interest in diffusion models, there may be some opportunity for those architectures to synthesize missing modalities. Diffusion models are a class of ML algorithms that can be used to analyze how information spreads or diffuses through a network.^94^ These models are commonly used to study the spread of diseases, ideas, or behaviors through social networks, but they can also be applied to other domains where information spreads through a network. One potential opportunity of using diffusion models for synthesizing missing modalities is that they can incorporate the structure of the network into the synthesis process, which can provide additional context and potentially improve the quality of the synthetic data.^95^^,^^96^^,^^97^ However, one challenge is that diffusion models may require the availability of a network structure, which may not always be available or may need to be constructed from other sources of data. Additionally, diffusion models can be computationally intensive, especially for large networks, and may require specialized algorithms and techniques to scale to these networks. Others have explored the use of meta-learning to generate missing modalities. Ma et al. introduced “multimodal learning with severely missing modality” (SMIL), which leverages Bayesian meta-learning to perturb the latent feature space so that the embeddings of a single modality can approximate ones of full modality. Notably, SMIL utilizes significantly fewer data to mitigate missing modality issues as compared to solutions that use generative networks.^98^ In a related field, multimodal translation has gained some attention in recent years as a potential method of both learning important semantic information from data modalities while simultaneously generating a potential missing modality.^88^

A larger parallel issue in C^4^ is modality incongruity, where sites may have heterogeneous data modalities available, and their local data consist of different combinations of modalities. For example, hospital A has omics, EHR, and CT data, whereas hospital B has omics, MRI, and wearable sensor data. In this scenario, hospitals A and B lack two modalities that the other has, but knowledge can still be derived from the complementary information of these missing modalities. This warrants the need for proper ways of learning personalizable information across multiple sites in the presence of modality incongruity. Zhao et al. propose an MMFL system in which clients can have unlabeled data of different modalities, and each client trains a deep canonical correlated autoencoder to model hidden representations between modalities. The local models are aggregated in the central server through multimodal federated averaging, where a supervised model is trained using the aggregated model’s encodings on an auxiliary dataset. While dealing with modality incongruity and the wealth of unlabeled data available at local clients, the framework of Zhao et al. does not take advantage of labeled data available at clients. Moreover, it requires an auxiliary dataset to be available at the central server, which is usually unrealistic in health care.^99^ The FedMSplit architecture leverages federated multitask learning (FMTL) to train on multimodal distributed data without necessitating uniform modalities across all clients. It utilizes a flexible multiview graph structure to dynamically identify correlations among multimodal client models. These client models are broken down into smaller blocks, each revealing a distinct perspective on client relationships. The multiview graph detects and disseminates client correlations as edge features, facilitating the learning of personalized yet globally connected multimodal client models. Despite being model-architecture neutral and efficient at processing nonindependent and identically distributed (IID) data, FedMSplit cannot utilize unlabeled data from sites, constraining its usability in healthcare scenarios where labeled data are limited.^100^

A critical concern when addressing modality incongruity in C^4^ is the preservation of data connectivity. To align the semantic feature space between different data modalities, it is essential that no block on the patchwork is isolated, meaning a particular modality must not be present exclusively at one site (see Figure 2). Isolated blocks may hinder the identification of complementary information between modalities, thus impacting the efficacy of the learning framework. Although most current investigations do not face significant data connectivity issues due to the limited number of modalities involved, real-world systems that incorporate 10 or more modalities are more susceptible to such challenges. For instance, site A may possess accelerometer data from a wearable sensor, which is unavailable at other sites. This isolation restricts the ability to determine how the accelerometer data could complement other modalities. Administratively, this could necessitate a decision on whether to include site A in the C^4^ setting. In scenarios where a C^4^ setting has isolated blocks of data, external sources of connective information will be needed to properly perform training. One solution for connecting these isolated blocks is through multimodal generation via LLMs. LLMs excel at transfer learning and domain adaptation, which enables them to transfer knowledge from one domain or task to another with minimal labeled data. This capability can be leveraged to establish connections between isolated data blocks and adapt models to site-specific tasks. The general-purpose representations learned by LLMs can be fine-tuned on specific medical tasks or modalities, adapting the models to the unique requirements and nuances of healthcare applications. This process of fine-tuning can help LLMs learn to better connect isolated data blocks and facilitate the extraction of complementary information across modalities. Recent advancements in this field include the development of Generative Pre-trained Transformer 4 (GPT-4, HuggingGPT, and PalmMED, among others.^79^^,^^101^^,^^102^ These models demonstrate the potential for LLMs to support solutions in C^4^ by addressing modality incongruity and data connectivity challenges. Necessary connective data can also be extracted from large-scale knowledge graphs such as the Integrative Biomedical Knowledge Hub and the Clinical Knowledge Graph.^103^^,^^104^ Biomedical knowledge graphs can be a valuable resource for connecting disparate datasets in C^4^ solutions as well. These graphs are large-scale, structured networks of biomedical information that can be used to represent and link various concepts, entities, and relationships in the domain of health and medicine. By using knowledge graphs, researchers can extract external information that can be used to connect different datasets in a C^4^ setting. For example, knowledge graphs can be used to identify shared concepts or entities between different datasets, such as specific diseases, drugs, or genes. This information can be used to map the data from different datasets onto a common ontology or feature space, allowing the data to be more easily combined and used for training ML models. Additionally, knowledge graphs can be used to provide contextual information about the data, such as the relationships between different entities or the attributes of specific concepts. This can help improve the accuracy and interpretability of the ML models and can also support the development of more complex and sophisticated models that can better capture the complex relationships and dynamics of health and disease. The use of external information extracted from biomedical knowledge graphs can be a valuable approach for connecting disparate datasets. To do so, knowledge graphs need to be multimodal and need to be able to adeptly link information across modalities.^105^^,^^106^ The idea of generating connective blocks is depicted in Figure 4.

### Interpretation of models: Solutions need to be explainable for healthcare application

There is significant interest in understanding the complex cross-modal associations in diagnostic decisions to further uncover hidden disease mechanisms, facilitate understanding of the disease, and build trust in statistical models. In clinical decision making, the interpretability of models is especially important, as several checks and balances need to be established when generating diagnoses or providing recommendations. Interpretability should seek to address both modality-specific contributions and intermodality interaction contributions.^107^ Simple approaches that have seen some success involve treating each modality separately when determining the post hoc interpretability of the modality. Han et al. utilized a multimodal network to estimate postoperative pain, employing Shapley additive explanations (SHAP) for model explanations on the fused multimodal space. However, it falls short in providing clinically evaluable details about each modality’s contribution.^108^ Furthermore, such methods are constrained in detailing the contribution of complementary information crucial for the performance of multimodal models. Others have developed modality-agnostic methods through post hoc model interpretation. DIME (fine-grained interpretations of multimodal models via disentangled local explanations) provides explanations for model predictions by disentangling the contributions of a model into those that are due to unimodal contributions and multimodal interactions. By doing so, clinicians can identify what facets of the overall model a prediction is based on. Although DIME is model agnostic, it has only been shown to work on models that provide discrete outputs. Moreover, as the number and diversity of modalities increase, the cost of disentanglement and interaction explanation becomes exponentially higher.^109^

Other multimodal networks are intrinsically interpretable through model design. These include graph-based fusion techniques, multimodal explanation networks, neurosymbolic reasoning, or attention-based methods.^87^^,^^110^^,^^111^ These approaches individually focus on building interpretable components for either modality or modality interaction. Attention-based approaches, where weights are assigned to different input features, have attracted significant attention recently. However, the explanatory power of these mechanisms is questionable since there is often a lack of association between gradient mappings and attention weights.^112^^,^^113^^,^^114^ In general, these methods suffer from only working due to careful model design and are limited to providing explanations only on specific modalities. Lack of access to cross-client data limits several interpretation mechanisms in their ability to provide both global and local explanations.

Causality is a crucial aspect in enhancing the interpretability of models because causal relationships are inherently comprehensible to humans. Causal ML facilitates the investigation of a system’s response to an intervention (e.g., outcomes given a treatment in the healthcare domain). Quantifying the effects of interventions (causal effects) enables the formulation of actionable decisions while maintaining robustness in the presence of confounders.^115^ In the context of C^4^, multimodal data can serve as proxies for unobserved confounders, thereby improving the accuracy of causal effect estimation.^116^ Addressing the missingness of modalities is a vital consideration for this objective. Furthermore, estimating the heterogeneous causal effects across different sites presents a challenge for causal ML within the C^4^ setting.^117^

### Distribution drift: Heterogeneity between sites needs to be accounted for in C^4^

Considering the regional disparities among participating healthcare facilities, the distribution of data across clients can vary significantly. This not only results in sample heterogeneity and non-IID data dispersed across sites but also leads to potential variations in the relationships between input features from one site to another. In C^4^, the existence of multiple modalities and the potential absence of some modalities at specific locations further exacerbate the challenge of addressing distribution drift. For instance, positron emission tomography (PET) scans may be captured using distinct scanners and protocols at various sites, leading to differences in image resolution, size, and interslice spacing. Consequently, the relationships between these PET scans and their corresponding site-specific EHR data may differ. Distribution drift has been identified as a primary factor contributing to model performance degradation and unfairness in multidomain settings, necessitating additional communication rounds for MMFL systems to achieve convergence.^118^

Domain generalization is one ML area that addresses distribution drift. Specifically, domain generalization presumes the existence of data from multiple source sites. Several methods have been proposed for training a model utilizing multisource data, ensuring generalizability to any unseen site.^119^ Muandet et al. suggested learning an invariant transformation of the input by minimizing dissimilarity across domains while preserving the functional relationship between input and output variables.^120^ Furthermore, the authors provided a learning-theoretic analysis demonstrating that reducing dissimilarity enhances expected generalization in new domains. This objective can also be accomplished through adversarial training.^121^ Additionally, some studies have focused on learning an invariant transformation of the conditional distribution of the input given the outcome class, rather than the input itself.^122^ These approaches prove effective in addressing conditional shifts across sites.

In addition to the aforementioned methodologies, a series of techniques known as domain invariant learning have been proposed to address domain generalization. Invariant risk minimization (IRM), introduced by Arjovsky et al., aims to reduce the effect of spurious, or noncausal, properties within different sets of training data. In a setting in which training data are split into multiple separate environments with their own site-specific biases, IRM promotes the learning of features that are stable across sites.^123^ More specifically, IRM starts by defining a set of tasks that the model needs to learn. Each task is associated with a different distribution of input data, and the goal is to learn a model that performs well on all of these tasks. The model is trained by minimizing a loss function that combines an ML model’s standard loss with a penalty term that encourages invariance across the different tasks. This penalty term is designed to measure the difference between the model’s predictions on two different tasks, and it is minimized when the model produces similar outputs for similar inputs, regardless of the task. Zare et al. introduced ReConfirm, which extends the IRM framework by accounting for class conditional variants and shows significant improvements over traditional trained ML models on medical data.^124^ In C^4^, IRM can be used to train a model that integrates information from multiple datasets that are distributed across separate sites and contain different modalities. Specifically, IRM can be used to learn a set of features that are consistent across different datasets, even if they contain different modalities or have different patient populations. By doing so, IRM can promote the generalization of the model to new data modalities and patient populations, thereby reducing distribution drift across sites.

In recent years, there has been a push to generate personalized, globally correlated models to mitigate drift across clients and data modalities. Personalized federated models are grouped into two categories: global model personalization and local-level personalization.^14^ Global model personalization trains a single global model that is subsequently personalized for each client through local adaptation. One implementation of global model personalization is Per-FedAvg and its extension pFedMe.^16^^,^^125^ Per-FedAvg uses model-agnostic meta-learning (MAML) to formulate FedAvg into developing an initial global model that performs well on heterogeneous clients, with only a few steps of gradient descent. Chen and Li propose hierarchical gradient blending (HGB), which adaptively calculates an optimal blending of modalities to minimize overfitting and promote generalization. HGB is task and architecture agnostic and shows promise in mitigating the lack of generalization in MMFL. However, initial implementations of HGB are not able to make the most use of complementary information between modalities.^126^

Local-level personalization can be further divided into two categories: architecture-based and similarity-based approaches. Architecture-based approaches enable personalization by designing different models for each client, whereas similarity-based approaches seek to identify client relationships and provide related clients with similar models.^15^ FedMD, an architecture-based approach, allows for the creation of personalized, architecture-agnostic models at clients through the use of transfer learning and knowledge distillation. Although the architecture-agnostic aspect of FedMD potentially allows the incorporation of differing modalities at different clients, FedMD requires a public dataset, which is infeasible in many healthcare scenarios.^127^ Lu et al. use FedAP to mitigate heterogeneity across clients by calculating the similarity between clients based on batch normalization weights. FedAP creates personalized models with less communication costs and has been evaluated on several healthcare datasets.^128^ An extension of these classes of architectures, FedNorm utilizes the minibatch normalization (MN) technique, an extension of using batch normalization, to create personalized models in the presence of data heterogeneity and to combat modality incongruity. The framework normalizes feature information by modality before distribution across all clients. FedNorm allows clients to have a mix of modalities while simultaneously combating data heterogeneity by building personalized models. However, FedNorm has shown success only when the modalities available are all of the same data type (e.g., PET scan and MRI—both images).^129^ A widely used strategy for local-level personalization involves federated multitask learning. Here, a model concurrently executes various related tasks, utilizing domain-specific knowledge from different clients. FMTL has shown promise in building models in federated settings with the MOCHA and FedAMP algorithms with techniques such as FedMSplit.^126^^,^^130^^,^^131^ In a recent study, Collins et al. introduce FedRep, a novel FL framework and algorithm, for the purpose of learning shared representations across distributed clients and unique local heads for each client. FedRep addresses the challenge of biases in current ML models by incorporating data from different modalities and sources, resulting in a shared feature representation that can be applied to a variety of tasks. FedRep’s ability to learn shared low-dimensional representations among data distributions makes it useful for meta-learning and multitask learning in C^4^ settings.^132^ Table 1 shows the advantages and disadvantages of many of the methods described above.

**Table 1:** Pros and cons of various methods that have been proposed to mitigate distribution drift in a C^4^ setting

Description	Pros	Cons	Implementation
MMFL system^99^	Support for multimodal data Can cope with modality incongruity Uses unlabeled data at local clients	Does not take advantage of labeled data available at clients Requires an auxiliary dataset with labels at the central server Dependence on alignment information	–
FedMSplit^100^	Uses FMTL on distributed data Uses a flexible multiview graph to identify correlations among client models Model-architecture neutral Efficient with non-IID data	Cannot use unlabeled data from sites Can expose local models to neighboring clients during aggregation Untested on real large-scale scenarios	–
Per-FedAvg and pFedMe^16^^,^^125^	Uses MAML for FedAvg to create an initial global model Effective for heterogeneous clients after few gradient descent steps	pFedMe can have high computational complexity due to use of Moreau envelopes Need sufficient data at each client Could have slow convergence rates	https://github.com/KarhouTam/Per-FedAvg https://github.com/CharlieDinh/pFedMe
HGB^126^	Task and architecture agnostic Can minimize overfitting and promote generalization	Cannot efficiently use complementary information between modalities	–
FedMD^127^	Allows for architecture-agnostic personalized models at clients	Requires a public dataset and initial training on it Complex communication module	https://github.com/Tzq2doc/FedMD
FedAP^128^	Mitigates client heterogeneity Lower communication costs Adaptive partitioning, which allows adaptation to non-IID data	May not be effective in all data heterogeneity scenarios Dependence on the accuracy of partitioning	https://github.com/KarhouTam/FL-bench
FedNorm^129^	Normalizing feature information by modality before distribution Allows mixed modalities per client	Effective only when modalities are of the same data type, (e.g., X-ray, ultrasound)	–
FedRep^132^	Learns shared low-dimensional representations among data distributions Generates a shared feature representation suitable for many tasks	Limitations with widescale applicability	https://github.com/lgcollins/FedRep

Healthcare data’s rapid evolution can cause concept drift, where models in C^4^ may become outdated over time.^133^ It is imperative to adapt these models to such distribution changes for sustained real-world application. Current solutions involve drift detection, followed by timely clustering or other adaptive strategies.^118^ Continual learning offers another approach, allowing models to learn incrementally without forgetting past tasks.^38^ Continual learning methods include frequent monitoring, fine-tuning, and even retraining a model over time. Despite its promise, practical challenges such as catastrophic forgetting—a decline in performance due to new information—plague continual learning. Hence, online training methods that utilize only new data may be more suited for health care. Implementing these models clinically also demands addressing the lack of standard assessment methods. Validation of these models needs to encompass factors such as the collection process for new data, the automated organization or labeling of new data, knowledge transfer between new and original data, and the overall performance of the model after incorporating data, while ensuring that no catastrophic interference occurs.

### Communication efficiency: Techniques to minimize lag due to communication are instrumental for C^4^ solutions

Communication is a key bottleneck to consider when developing methods for C^4^. This is because a C^4^ setting could include a massive number of sites or individual patient silos, and communication in the system can be slower than local computation by many orders of magnitude. With the integration of multiple data modalities, computation time increases because many current MML techniques require significant amounts of preprocessing and/or communication to integrate. Therefore, real-world C^4^ solutions will have to rely on communication-efficient methods.

A category of methods emphasizes optimizing local updating processes. These permit a variable number of parallel local updates on each machine during each communication round. The primary aim of these local updating methods is to minimize the total communication rounds. Guha et al. introduce one-shot FL, where the central server requires only a single round of communication to learn a global model through the use of ensembling and model distillation.^134^ Zhou et al. expand one-shot FL through data distillation one-shot FL, in which each client distills their data to be sent to the central server, where a global model is trained.^135^ COMMUTE utilizes transfer learning and distance-based adaptive regularization to create a one-shot multisite risk prediction framework. Although the method mitigates the effects of data heterogeneity across sites, it limits all clients to using the same set of features. Moreover, its performance with complex and deep model architectures remains unknown.^136^

Another class of methods that has seen success in decreasing communication costs is model compression, which includes sparsification, subsampling, and quantization. Zhang et al. introduced dynamic fusion-based FL to choose participating clients according to local performance, thereby improving communication efficiency. They applied this method to predict COVID-19 across secure nodes and showed performances comparable to and/or higher than FedAvg on different facets.^137^ Recently, decentralized training has garnered a great deal of attention for its ability to increase communication efficiency. Although standard FL settings require a central server for connecting all remote devices and performing aggregations, decentralized FL systems provide an alternative when communication to the server becomes a bottleneck, especially when operating in low bandwidth or high-latency networks.^4^ Table 2 presents advantages and disadvantages of the various communication methods.

**Table 2:** Pros and cons of various communication methods that have been proposed

Method	Description	Pros	Cons	Implementation
One-shot FL^134^	Introduced by Guha et al., it requires only a single communication round to the central server to learn a global model via ensembling and model distillation	Minimizes total communication rounds	May not be ideal for all data types and scenarios	–
DOSFL^135^	An expansion of one-shot FL by Zhou et al.; each client distills their data before sending it to the central server for global model training	Optimized for minimized communication	Nuances of data handling and performance in diverse settings need more exploration	–
COMMUTE^136^	Uses transfer learning and adaptive regularization for a one-shot multisite risk prediction framework	Mitigates data heterogeneity effects	Forces all clients to use the same set of features Performance with deep model architectures is uncertain	https://github.com/biostat-duan-lab/multiTL
Dynamic fusion-based FL^137^	Introduced by Zhang et al., it selects participating clients based on local performance to improve communication efficiency; applied to predict COVID-19 across secure nodes	Increases communication efficiency Comparable or better performance than FedAvg in some scenarios	Requires dynamic monitoring and more computational resources at the client end	https://github.com/GalaxyLearning/GFL
Decentralized training^4^^,^^145^	Does away with the central server, allowing for direct communication between remote devices	Enhances communication efficiency, especially in low bandwidth or high-latency situations	May be prone to security issues and requires robust peer-to-peer networking Data synchronization could be challenging	https://github.com/pguijas/p2pfl

DOSFL, data distillation one-shot FL.

### Privacy: C^4^ solutions should have infrastructure and methods to ensure client privacy

Data privacy is of the utmost importance in health care, particularly when it comes to training ML models. Patient data are highly sensitive and must be protected to maintain trust and confidentiality. However, ML models require large amounts of data to be effective, creating a tension between privacy and innovation. Therefore, developing privacy-preserving ML techniques for health care can help mitigate these concerns and enable the development of accurate models while preserving patient privacy.^7^^,^^8^ Carlini et al. have shown that unwitting memorization of neural networks from the training dataset may reveal personally identifiable information.^138^ Moreover, models themselves may contain intellectual property (IP), and the learned parameters of the models can reveal valuable information about the model’s architecture, design, and functionality, which could be used by others to replicate or reverse-engineer the model without the owner’s permission. If the information within the datasets is leaked through ML models, it could not only harm privacy but also undermine trust in such collaborative implementations.^139^

In health care, privacy-preserving methods are essential for ensuring the privacy of customer information, as data used to train ML models could be compromised and exploited through an attack from adversaries. With the introduction of FL methods to mitigate issues with data heterogeneity and data sharing challenges, these aforementioned attacks have become more viable. Secure multiparty computation (SMPC), differential privacy, and homomorphic encryption were introduced to prevent malicious attacks.^139^ Each of these methods has its advantages and shortcomings. SMPC and homomorphic encryption are computationally costly and require complex infrastructure to maintain.^140^ With differential privacy, the performance of ML models is usually compromised for increased security.^141^ Moreover, although differential privacy and other obfuscation techniques have shown some promise in unimodal data, there are adaptation issues when working in an MML setting. These methods are often fine-tuned for specific scenarios or model algorithms. The diversity of data modalities generates different definitions of differential privacy algorithms, which leads to difficulties in unifying them into one algorithm.^142^

Recently, the large-scale use of blockchain has provided researchers with another method for increasing the security of distributed systems. Chang et al. designed a blockchain-based FL framework for medical IoT devices, which utilizes differential privacy and gradient-verification protocols to catch poisoning attacks. When tested on the task of diagnosing diabetes based on EHR data, their architecture is able to limit the success of poisoning attacks to less than 20%.^143^ Another framework, proposed by Rehman et al., uses blockchain and an intrusion detection system to detect malicious activity during model training within a federated healthcare network. The end-to-end system allows for models to be developed on several modalities, ranging from medical IoT data to medical images, and gives physicians the ability to monitor patient risk for diseases in real time. Although currently limited in its computational complexity, the framework shows promise in providing a system for medical organizations to develop risk prediction models based on multimodal data.^144^

Swarm learning (SL) uses blockchain technology to combine decentralized hardware infrastructures to securely onboard clients and dynamically generate a global model whose performance is comparable to models trained when all of the data are pooled. Through the use of blockchain technology, SL is able to mitigate the harm of dishonest participants or adversaries attempting to undermine the network. SL has demonstrated its utility in preliminary applications, predicting conditions such as COVID-19 and leukemia where clients possess non-IID data. Despite decentralization strengthening network resilience to attacks and data heterogeneity, SL’s absence of central aggregators reduces some capabilities inherent in other frameworks. Moreover, the current implementations of SL may be affected by latency between clients, slowing calculation transportations.^145^

### Fairness: Solutions should promote fairness across clients

One major challenge of FL, which becomes even more difficult in C^4^, is achieving collaborative fairness among participating clients. Each client’s contribution to the central model is usually far from equal due to various reasons, with the primary reason being distributional discrepancies across different clients. In certain scenarios, some clients may be negatively affected through distributed learning.^15^ As ML models are deployed in increasingly important applications, ensuring that the trained models do not discriminate against sensitive attributes has become another critical factor for FL. In general, fairness falls under two categories: (1) performance fairness, where every client sees a performance increase from participating and (2) collaboration fairness, where participants with higher contributions receive higher rewards or incentives. These incentives can include reputation, monetary compensation, or additional computational infrastructure, among others.^146^^,^^147^

To ensure performance fairness, Li et al. propose a q-Fair FL framework to achieve an improved uniform accuracy distribution across participants at the cost of model performance.^147^ q-Fair FL utilizes a novel optimization technique that reweights local objectives, which was inspired by resource allocation strategies in wireless networks.^142^ Agnostic federated learning optimizes a model for any target distribution formed by a mixture of clients’ distributions, forcing the model not to overfit to any particular client.^148^ Hao et al. propose Fed-ZDAC and Fed-ZDAS, which utilize zero-shot data augmentation (generating synthetic data based only on model information rather than sample data points) on underrepresented data to decrease statistical heterogeneity and encourage uniform performance across clients.^149^ Other methods have utilized multitask FL and other personalization techniques to achieve performance fairness by mitigating the presence of data heterogeneity, which is often the root cause of nonuniform performance.^15^^,^^150^

Collaborative fairness is essential when there are discrepancies in contributions between clients. These contributions can vary due to data volume, data quality, computation power, and the potential risks that each client takes by participating in an C^4^ solution. A fair collaborative environment is one in which each participant receives a reward that fairly reflects its contribution to C^4^. When developing collaborative fair environments, measurement of contribution, reward for contribution, and distribution of reward all need to be determined. The federated learning incentivizer (FLI) was proposed as a payoff-sharing scheme to achieve contribution and expectation fairness. FLI is formulated to work with any definition of contribution and cost but is primarily used for monetary rewards, which is not the norm in healthcare scenarios.^151^ The robust and fair FL (RFFL) method assigns superior-performing models to clients who contribute more significantly. RFFL determines a “reputation” score for each client to signify their level of contribution. Clients falling below a specified contribution threshold are subsequently excluded from the process.^152^ Cui et al. propose the concept of collaboration equilibrium, where clients are grouped such that no individual client could gain more in another configuration.^146^ They employ a Pareto optimization framework and benefit graphs to create clusters of clients that reach this equilibrium. Although this approach exhibits potential for achieving collaborative fairness, it necessitates all local clients’ consent to construct a benefit graph by a neutral third party before the initiation of model training.^146^

## Future directions

Addressing C^4^ remains a complex but rewarding endeavor. The development of solutions that enable data integration across multiple sites and modalities could revolutionize health care. Such solutions could allow researchers to amalgamate data from various datasets, thereby providing a more comprehensive and precise perspective on health and disease. This would support the enhancement of diagnostic, predictive, and therapeutic tools. However, these potential solutions come with challenges, including communication efficiency, privacy, and fairness. Overcoming these hurdles requires continuous exploration and development of new methods, technologies, and research into fairness and bias. The goal is to ensure equitable and accurate results. Ultimately, successfully addressing C^4^ could lead to innovative tools for diagnosing, predicting, and treating health conditions, thereby transforming the healthcare landscape.
